# Supplementary material for: How is depth of anaesthesia assessed in experimental pigs? A scoping review
Source: PLoS One. 2023 Mar 23;18(3):e0283511. doi: 10.1371/journal.pone.0283511 (PMC10035875; doi:10.1371/journal.pone.0283511)
Supplement: S1 File — (DOCX) [file pone.0283511.s001.docx]

**Protocol**

The following report represents the protocol used for performing the scoping review entitled: “Depth of anaesthesia assessment in experimental pigs: A scoping review” and was developed following the PRISMA-P guidelines (adapted to the scoping review needs).

**Authors contacts**

Alessandro Mirra, Section of Anaesthesiology and Pain Therapy, Department of Clinical Veterinary Medicine, Vetsuisse Faculty, University of Bern, Bern, Switzerland [alessandro.mirra@vetsuisse.unibe.ch](mailto:alessandro.mirra@vetsuisse.unibe.ch) (corresponding author)

Ekaterina Gamez Maidanskaia, Section of Anaesthesiology and Pain Therapy, Department of Clinical Veterinary Medicine, Vetsuisse Faculty, University of Bern, Bern, Switzerland [ekaterina.gamez@vetsuisse.unibe.ch](mailto:ekaterina.gamez@vetsuisse.unibe.ch)

Luís Pedro Gomes do Carmo, Veterinary Public Health Institute, Department of Clinical Research und Public Health (DCR-VPH), Vetsuisse Faculty, University of Bern, Bern, Switzerland AND Norwegian Veterinary Institute, Ås, Norway. [luis.gomesdocarmo@vetsuisse.unibe.ch](mailto:luis.gomesdocarmo@vetsuisse.unibe.ch)

Luis.Pedro.Carmo@vetinst.no

Olivier Levionnois, Section of Anaesthesiology and Pain Therapy, Department of Clinical Veterinary Medicine, Vetsuisse Faculty, University of Bern, Bern, Switzerland olivier.levionnois@vetsuisse.unibe.ch

Claudia Spadavecchia, Section of Anaesthesiology and Pain Therapy, Department of Clinical Veterinary Medicine, Vetsuisse Faculty, University of Bern, Bern, Switzerland [claudia.spadavecchia@vetsuisse.unibe.ch](mailto:claudia.spadavecchia@vetsuisse.unibe.ch)

**Authors contributions**

AM (guarantor of the review) developed the search and screening strategy, performed the literature research, and wrote the manuscript.

EGM performed the screening, read and amended the manuscript.

LPGC developed the search and screening strategy, read and amended the manuscript.

OL developed the search strategy, read and amended the manuscript.

CS developed the search strategy, read and amended the manuscript.

**Support**

The review was financially supported by the Section of Anaesthesiology and Pain Therapy, Department of Clinical Veterinary Medicine, Vetsuisse Faculty, University of Bern, Bern, Switzerland.

1. **Introduction and objective**

In the field of Veterinary Anesthesiology, multiple methods are available to assess the patients’ depth of anesthesia. Their relevance in guiding the veterinary anesthetist during surgical and diagnostic procedures is undeniable. Acknowledging the importance of such tools, we aimed at investigating and synthetizing the information available on the evidence that sustains or contradicts the use of such evaluation methods in pigs.

Such process will allow:

1. To identify the methods that have been investigated to assess DoA in pigs;
2. To investigate and summarize the evidence that sustains or contradicts the use of DoA indicators present in the literature and adopted in pigs and minipigs undergoing general anaesthesia;
3. To identify and analyse knowledge gaps.

Considering the scope of our review, as well as the abovementioned objectives, we deemed a scoping review the most adequate process. This was chosen in detriment of a systematic review based on the selection criteria elaborated by Munn et al. (2018). In short:

- We have a broad scope that encompasses multiple methods, substances and study types;
- We did not have a narrower research question that would fit best a systematic review;
- We aim at scoping the available evidence and examine how research is done to support the use of these methods;
- We intend to identify and analyze knowledge gaps;
- Despite the fact that our results might be of relevance in the context of the clinical decision-making process, we are not focusing on a single assessment method.

1. **Title**

How is depth of anaesthesia assessed in experimental pigs? A scoping review

1. **Research question**

“Is there scientific evidence of the usefulness of the methodologies commonly employed to assess depth of anaesthesia (DoA) in pigs?”.

Definitions:

1. Pig = Sus/Sus scrofa domesticus.
2. Depth of anaesthesia = degree of depression of the central nervous system;
3. Scientific evidence = methods have been validated and/or a large amount of data as well as a wide experience of the scientific community support their usage.
4. **Search Terms**

Refer to the Word document:

- “S2 File”

1. **Inclusion/ Exclusion criteria**

**Population**:

| ***Inclusion*** | ***Exclusion*** |
| --- | --- |
| Pig; Minipig | Other animal species |

Pig and minipigs have been included because they are among the most used large animal laboratory species in translational research. In spite of this, species-specific monitoring strategies have not been developed.

**Interest:**

| ***Inclusion*** | ***Exclusion*** |
| --- | --- |
| Depth of anaesthesia monitoring strategies used/declared/investigated | No depth of anaesthesia monitoring strategies used/declared/investigated |

When inducing general anaesthesia, it is necessary to be able to objectively judge the central nervous system depression (depth of anaesthesia). Indeed, general anaesthesia leads to an impossibility of having feedback from the animal (e.g. behavioural responses, modification of normal behaviour), and it is of paramount importance to find monitoring strategies that highly correlate with the level of central nervous system depression.

**Context:**

| ***Inclusion*** | ***Exclusion*** |
| --- | --- |
| Animal undergoing general anaesthesia | Animal awake or undergoing sedation |

In order to perform translational studies, general anaesthesia is often required. While more information is present in the literature regarding how to ensure animal welfare in an awake status, almost no information is available in a general anaesthesia status.

**Others**

***Studies:*** Original researches (no reviews, case reports, letters to the Editor)

***Time:*** No time limitation.

***Languages:*** No language limitation.

1. **Search Strategy**

Electronic databases: 3 databases (PubMed, Embase, CAB Abstract).

1. **Study Selection**
2. Preliminary screening: in collaboration with the systematic review services coordinator of the University of Bern, the main author (AM) will perform a three phases procedure to create a list of depth of anaesthesia monitoring indicators used in pigs, to then include them in a final search string (see the Word document “Search strings”);
3. Abstract selection (Title + Abstract) (AM, EGM).

Questions to be answered for abstract selection:

1. Is the paper an original research? (no=excluded)
2. Is the studied animal a pig/minipig? (no=excluded)
3. Is it an in vivo study? (no=excluded)
4. Is general anaesthesia performed? (no=excluded)
5. Is a DoA indicator reported (even if not specifically declared to be used to assess DoA)? (no=excluded). (This question was added at a later stage).

1. Full-text selection (AM + CS and OL in case of doubts).

Papers will be excluded if:

1. the authors did not use any indicator to assess DoA;
2. the DoA indicators are not reported for at least two well distinct time points and/or drug combinations and/or drug concentrations and/or physiological conditions and/or surgical interventions;
3. unconsciousness was not induced by drug administration (e.g., electricity, carbon dioxide);
4. only a minimum alveolar concentration (MAC) extrapolated from other studies was used to guide anaesthetic administration;
5. the paper is a duplicate, or at least one of the five abstract inclusion criteria is not fulfilled;
6. only the abstract is available.
7. **Data Extraction**
8. Data from abstract will be extracted separately by two investigators (AM, EGM) and inserted in two different Excel files. Differences will be discussed and resolved by consensus.
9. Data from full texts will be extracted by one investigator (AM) and inserted in an Excel file. Doubts will be discussed and resolved by consensus with the co-authors (CS, OL).

The DoA indicators found will be then grouped in two categories:

A: indicators specifically investigated as method to assess DoA;

B: indicators used to assess DoA, which outcomes were only described and not investigated as method to assess DoA.

For both categories, we are aiming to extract the following information from the relative papers:

- first author’s surname;
- year of publication;
- journal;
- indicator studied.

Moreover, for papers describing indicators of category A, further information will be retrieved:

- if a statistical analysis was performed;
- essential results;
- animals’ weight;
- animals’ age;
- animals’ breed;
- animals’ sex;
- number of animals included in the study;
- main anaesthetic drug used.

1. **Quality assessment**

The studies that will be included in the research are expected to be heterogeneous and often not focusing of depth of anaesthesia monitoring. Thus, a quality assessment will not be feasible for the aim of the present study.

We expect a wide difference also in the number of studies performed for each single monitoring indicator included in our research string. For some of them, quantitative data and more precise data regarding their usefulness in monitoring depth of anaesthesia are expected. In that case, some quantitative data extraction will be performed.

As a sub-analysis, the monitoring tools used in the papers included in the full text screening will be reported.

1. **Data synthesis**

Data synthesis will be performed according to the extracted data. These results will be analyzed and critically assessed.

The outcomes of our study will be summarized in a paper format. As previously mentioned, a scoping review was deemed the most appropriate methods to attain our objectives. This evidence synthesis method assures a transparent, systematic and reproducible method to scope the literature on a given topic.

We will follow and write the paper according to the PRISMA Extension for Scoping Reviews (PRISMA-ScR) guidelines, as we believe this will increase the quality of our study.
