# Supplementary material for: How is depth of anaesthesia assessed in experimental pigs? A scoping review
Source: PLoS One. 2023 Mar 23;18(3):e0283511. doi: 10.1371/journal.pone.0283511 (PMC10035875; doi:10.1371/journal.pone.0283511)
Supplement: S2 File — (DOC) [file pone.0283511.s002.doc]

**MEDLINE**

Database: Ovid MEDLINE(R) and Epub Ahead of Print, In-Process & Other Non-Indexed Citations, Daily and Versions(R) <1946 to September 21, 2022>

Search Strategy:

--------------------------------------------------------------------------------

1 exp Swine/

2 exp Anesthesia/

3 exp anesthetics/ or exp "hypnotics and sedatives"/ or exp narcotics/

4 exp Intraoperative Awareness/ or exp Awareness/

5 exp H-Reflex/ or exp Reflex/

6 exp Transcutaneous Electric Nerve Stimulation/

7 exp Eye Movements/

8 exp Pupil/

9 exp Shivering/

10 exp Movement/

11 exp Heart Rate/

12 exp Respiratory Rate/ or exp Blood Pressure/

13 exp Hemodynamics/

14 exp Electroencephalography/

15 exp Consciousness Monitors/

16 exp Consciousness/

17 exp Entropy/

18 exp Spectroscopy, Near-Infrared/

19 exp Electrocorticography/

20 exp Evoked Potentials/

21 exp Nociception/

22 exp Monitoring, Intraoperative/ or exp Intraoperative Neurophysiological Monitoring/

23 exp Audiometry, Evoked Response/ or exp Laser-Evoked Potentials/

24 exp Visual Analog Scale/

25 (pig? or piglet? or swine? or hog? or sus scrofa or minipig? or mini-pig? or sow? or boar? or piggy or piggies or porker? or shoat?).ab,kw,ti.

26 (an?esthetic? or an?esthesia or dissociative? or narcosis or narcotic? or hypnosis or hypnotic?).ab,kw,ti.

27 "reflex*2".ab,kw,ti.

28 (pinch? or pinching? or clamp? or clamping?).ab,kw,ti.

29 prick*.ab,kw,ti.

30 "supramaximal stimul*".ab,kw,ti.

31 (eye? adj2 rotation?).ab,kw,ti.

32 (eye? adj2 position*3).ab,kw,ti.

33 nystagmus.ab,kw,ti.

34 (pupil? or tone or shiver* or tremor? or movement? or twitch*).ab,kw,ti.

35 "physiological parameter?".ab,kw,ti.

36 "heart rate".ab,kw,ti.

37 "respiratory rate".ab,kw,ti.

38 "respiratory frequency".ab,kw,ti.

39 "blood pressure".ab,kw,ti.

40 "h?emodynamic change?".ab,kw,ti.

41 "bispectral index".ab,kw,ti.

42 "index of consciousness".ab,kw,ti.

43 entropy.ab,kw,ti.

44 "median frequency".ab,kw,ti.

45 "electrocorticogr*".ab,kw,ti.

46 "electro-corticogr*".ab,kw,ti.

47 "electroencephalo*".ab,kw,ti.

48 "electro-encephalo*".ab,kw,ti.

49 narcotrend.ab,kw,ti.

50 "burst suppression".ab,kw,ti.

51 "cerebral state index".ab,kw,ti.

52 "evoked potential?".ab,kw,ti.

53 (nociception or nociceptive or neuromonitor*).ab,kw,ti.

54 "neuro-monitor*".ab,kw,ti.

55 (evoked adj3 potential?).ab,kw,ti.

56 (evoked adj3 response?).ab,kw,ti.

57 (spectral adj3 voltage).ab,kw,ti.

58 (consciousness adj2 monitor*).ab,kw,ti.

59 "end tidal".ab,kw,ti.

60 "visual analogue scale".ab,kw,ti.

61 "simple descriptive scale".ab,kw,ti.

62 "suppression ratio".ab,kw,ti.

63 "end-expired concentration".ab,kw,ti.

64 (expired adj2 concentration).ab,kw,ti.

65 (fraction adj2 inspired).ab,kw,ti.

66 arousal/ or wakefulness/

67 (wakefulness or arousal or awareness or consciousness or unconsciousness).ab,kw,ti.

68 "spectr* analysis".ab,kw,ti.

69 "withdrawal response".ab,kw,ti.

70 "near infrared spectroscopy".ab,kw,ti.

71 exp Cough/

72 "cough*3".ab,kw,ti.

73 lacrimation.ab,kw,ti.

74 1 or 25

75 2 or 3 or 26

76 4 or 5 or 6 or 7 or 8 or 9 or 10 or 11 or 12 or 13 or 14 or 15 or 16 or 17 or 18 or 19 or 20 or 21 or 22 or 23 or 24 or 27 or 28 or 29 or 30 or 31 or 32 or 33 or 34 or 35 or 36 or 37 or 38 or 39 or 40 or 41 or 42 or 43 or 44 or 45 or 46 or 47 or 48 or 49 or 50 or 51 or 52 or 53 or 54 or 55 or 56 or 57 or 58 or 59 or 60 or 61 or 62 or 63 or 64 or 65 or 66 or 67 or 68 or 69 or 70 or 71 or 72 or 73

77 74 and 75 and 76

**EMBASE**

Database: Embase <1974 to 2022 September 22>

Search Strategy:

--------------------------------------------------------------------------------

1 exp pig/

2 exp anesthesia/

3 exp anesthetic agent/

4 exp hypnosis/

5 exp hypnotic agent/ or exp hypnotic sedative agent/

6 exp narcotic agent/

7 exp intraoperative awareness/ or exp awareness/

8 exp hoffmann reflex/

9 exp reflex/

10 exp nerve stimulation/

11 exp eye movement/

12 exp pupil/

13 exp shivering/

14 exp "movement (physiology)"/

15 exp heart rate/

16 exp breathing rate/

17 exp blood pressure/

Annotation: si potrebbe ridurre

18 exp hemodynamics/

19 exp electroencephalography/

20 exp consciousness/ or exp consciousness monitor/

21 exp entropy/

22 exp spectroscopy/

23 exp evoked response/

24 exp nociception/

25 exp neurophysiological monitoring/

26 exp intraoperative monitoring/

27 exp visual analog scale/

28 (pig? or piglet? or swine? or hog? or sus scrofa or minipig? or mini-pig? or sow? or boar? or piggy or piggies or porker? or shoat?).ab,kw,ti.

29 (an?esthetic or an?esthesia or dissociative? or narcosis or narcotic? or hypnosis or hypnotic?).ab,kw,ti.

30 "reflex*2".ab,kw,ti.

31 pinch?.ab,kw,ti.

32 pinching?.ab,kw,ti.

33 clamp?.ab,kw,ti.

34 clamping?.ab,kw,ti.

35 "prick*".ab,kw,ti.

36 "supramaximal stimul*".ab,kw,ti.

37 (eye? adj2 rotation?).ab,kw,ti.

38 (eye? adj2 position*3).ab,kw,ti.

39 nystagmus.ab,kw,ti.

40 pupil?.ab,kw,ti.

41 tone.ab,kw,ti.

42 "shiver*".ab,kw,ti.

43 tremor?.ab,kw,ti.

44 movement?.ab,kw,ti.

45 "twitch*".ab,kw,ti.

46 physiological parameter?.ab,kw,ti.

47 heart rate.ab,kw,ti.

48 respiratory rate.ab,kw,ti.

49 respiratory frequency.ab,kw,ti.

50 blood pressure.ab,kw,ti.

51 h?emodynamic change?.ab,kw,ti.

52 bispectral index.ab,kw,ti.

53 index of consciousness.ab,kw,ti.

54 entropy.ab,kw,ti.

55 median frequency.ab,kw,ti.

56 "electrocorticogr*".ab,kw,ti.

57 "electro-corticogr*".ab,kw,ti.

58 "electroencephalo*".ab,kw,ti.

59 "electro-encephalo*".ab,kw,ti.

60 narcotrend.ab,kw,ti.

61 burst suppression.ab,kw,ti.

62 cerebral state index.ab,kw,ti.

63 evoked potential?.ab,kw,ti.

64 nociception.ab,kw,ti.

65 nociceptive.ab,kw,ti.

66 "neuromonitor*".ab,kw,ti.

67 "neuro-monitor*".ab,kw,ti.

68 (evoked adj3 potential?).ab,kw,ti.

69 (evoked adj3 response?).ab,kw,ti.

70 (spectral adj3 voltage?).ab,kw,ti.

71 (consciousness adj2 monitor*).ab,kw,ti.

72 end tidal.ab,kw,ti.

73 visual analogue scale.ab,kw,ti.

74 simple descriptive scale.ab,kw,ti.

75 suppression ratio.ab,kw,ti.

76 end-expired concentration.ab,kw,ti.

77 (expired adj2 concentration).ab,kw,ti.

78 (fraction adj2 inspired).ab,kw,ti.

79 exp arousal/

80 exp wakefulness/

81 wakefulness.ab,kw,ti.

82 arousal.ab,kw,ti.

83 awareness.ab,kw,ti.

84 consciousness.ab,kw,ti.

85 unconsciousness.ab,kw,ti.

86 exp consciousness/

87 exp unconsciousness/

88 "spectr* analysis".ab,kw,ti.

89 withdrawal response.ab,kw,ti.

90 near infrared spectroscopy.ab,kw,ti.

91 "cough*3".ab,kw,ti.

92 lacrimation.ab,kw,ti.

93 exp coughing/

94 exp lacrimation/

95 1 or 28

96 2 or 3 or 4 or 5 or 6 or 29

97 7 or 8 or 9 or 10 or 11 or 12 or 13 or 14 or 15 or 16 or 17 or 18 or 19 or 20 or 21 or 22 or 23 or 24 or 25 or 26 or 27 or 30 or 31 or 32 or 33 or 34 or 35 or 36 or 37 or 38 or 39 or 40 or 41 or 42 or 43 or 44 or 45 or 46 or 47 or 48 or 49 or 50 or 51 or 52 or 53 or 54 or 55 or 56 or 57 or 58 or 59 or 60 or 61 or 62 or 63 or 64 or 65 or 66 or 67 or 68 or 69 or 70 or 71 or 72 or 73 or 74 or 75 or 76 or 77 or 78 or 79 or 80 or 81 or 82 or 83 or 84 or 85 or 86 or 87 or 88 or 89 or 90 or 91 or 92 or 93 or 94

98 95 and 96 and 97

**CAB ABSTRACT**

Database: CAB Abstracts <1984 to 2022 Week 37>

Search Strategy:

--------------------------------------------------------------------------------

1 (pig? or piglet? or swine? or hog? or sus scrofa or minipig? or mini-pig? or sow? or boar? or piggy or piggies or porker? or shoat?).ab,ti.

2 (an?esthetic? or an?esthesia or dissociative? or narcosis or narcotic? or hypnosis or hypnotic?).ab,ti.

3 "reflex*2".ab,ti.

4 pinch?.ab,ti.

5 pinching?.ab,ti.

6 clamp?.ab,ti.

7 clamping?.ab,ti.

8 "prick*".ab,ti.

9 "supramaximal stimul*".ab,ti.

10 (eye adj2 rotation?).ab,ti.

11 (eye adj2 position*3).ab,ti.

12 nystagmus.ab,ti.

13 pupil?.ab,ti.

14 tone.ab,ti.

15 "shiver*".ab,ti.

16 tremor?.ab,ti.

17 movement?.ab,ti.

18 "twich*".ab,ti.

19 physiological parameter?.ab,ti.

20 heart rate.ab,ti.

21 respiratory rate.ab,ti.

22 respiratory frequency.ab,ti.

23 blood pressure.ab,ti.

24 h?emodynamic change?.ab,ti.

25 bispectral index.ab,ti.

26 index of consciousness.ab,ti.

27 entropy.ab,ti.

28 median frequency.ab,ti.

29 "electrocorticogr*".ab,ti.

30 "electro-corticogr*".ab,ti.

31 "electroencephalogr*".ab,ti.

32 "electro-encephalogr*".ab,ti.

33 narcotrend.ab,ti.

34 burst suppression.ab,ti.

35 cerebral state index.ab,ti.

36 nociception.ab,ti.

37 nociceptive.ab,ti.

38 "neuromonitor*".ab,ti.

39 (evoked adj3 potential?).ab,ti.

40 (evoked adj3 response?).ab,ti.

41 (spectral adj3 voltage?).ab,ti.

42 (consciousness adj2 monitor*).ab,ti.

43 end tidal.ab,ti.

44 visual analogue scale.ab,ti.

45 simple descriptive scale.ab,ti.

46 suppression ratio.ab,ti.

47 end-expired concentration.ab,ti.

48 (expired adj2 concentration).ab,ti.

49 (fraction adj2 inspired).ab,ti.

50 wakefulness.ab,ti.

51 arousal.ab,ti.

52 awareness.ab,ti.

53 consciousness.ab,ti.

54 unconsciousness.ab,ti.

55 "spectr* analysis".ab,ti.

56 withdrawal response.ab,ti.

57 near infrared spectroscopy.ab,ti.

58 "cough*3".ab,ti.

59 lacrimation.ab,ti.

60 1 and 2

61 3 or 4 or 5 or 6 or 7 or 8 or 9 or 10 or 11 or 12 or 13 or 14 or 15 or 16 or 17 or 18 or 19 or 20 or 21 or 22 or 23 or 24 or 25 or 26 or 27 or 28 or 29 or 30 or 31 or 32 or 33 or 34 or 35 or 36 or 37 or 38 or 39 or 40 or 41 or 42 or 43 or 44 or 45 or 46 or 47 or 48 or 49 or 50 or 51 or 52 or 53 or 54 or 55 or 56 or 57 or 58 or 59

62 60 and 61

***************************
