# Supplementary material for: How is depth of anaesthesia assessed in experimental pigs? A scoping review
Source: PLoS One. 2023 Mar 23;18(3):e0283511. doi: 10.1371/journal.pone.0283511 (PMC10035875; doi:10.1371/journal.pone.0283511)
Supplement: Attachment — Submitted filename: RESPONSE TO REVIEWERS.docx [file pone.0283511.s006.docx]

***RESPONSE TO REVIEWERS***

Dear reviewers,

First, thank you very much for the time dedicated to our manuscript, as well as for the suggestions made to improve its quality.

Please find below the answers to your comments.

*Reviewer #1: well-conducted review of literature and analyze of different monitors and parameters. Constructive clear discussion to replace the contexte of avoid of awareness and memorization. the work takes place in the background of translational research using pig*

Thank you, we appreciate it.

*Reviewer #2:*

Thank you for the rigorous assessment of our manuscript and the suggestions provided.

*ABSTRACT

Line 45: It should be made clearer that the contrast results are not in relation to how useful the depth of anaesthesia indicators are but to what are the best depth of anaesthesia indicators.*

Thank you for your comment. Contrasting results have been found in relation to how useful the depth of anaesthesia (DoA) indicators were. We did not find papers specifically analysing which indicator was the best to assess DoA in pigs. The sentence has been modified to clarify better our results. *Lines 50-51: Maybe the authors should underline the lack of many high quality scientific studies and not only the lack of scientifically valid and reliable indicators of depth anaesthesia in pigs.*

Thank you for pointing it out. Due to the nature of the studies found and the type of review chosen, we did not analyse the quality of the papers, but rather screened their results. Our scoping review could be the base for future focused systematic reviews, that could look at the quality of single studies. We added a sentence in the discussion section *INTRODUCTION

Line 81: Why do they claim that the reliability of the results obtained from translational studies is questioned by the lack of valid depth of anaesthesia indicators? It should be explained in more detail.*

Thank you for spotting it. A sentence has been added to better explain the concept. The lack of knowledge on how to appropriately assess DoA brings to the difficulty of ensuring a correct DoA. This refers not only to the possible occurrence of intraoperative awareness per se, but also to all the correlated consequences (e.g., cardiovascular, respiratory, hormonal). This brings to the impossibility to differentiate, for example, physiological modifications occurring because of a certain surgical stimulation/treatment from those occurring due to an inappropriate DoA/intraoperative awareness. *Lines 87-88: Maybe the answer to this question is already elucidated in the lines 80-81, so the question should not address the usefulness of methodologies to assess DoA in pigs but should refer the best validated ones.*

This comment has been partially answered above. Unfortunately, in veterinary medicine and especially in pigs, no validated DoA indicators are present (as also shown by the present review). Thus, we did not want to look only for validated DoA indicators, but rather for all the methodologies used in pigs so far.

For example, we found that reaction to claw clamp has been widely used as sole DoA indicator, despite being inappropriate for this scope when used alone. Our review highlights how DoA assessment has not been always done in a correct way in pigs, and how really few steps toward a better DoA assessment have been performed in the last decades. *Lines 89-91: It is not necessary to describe the differences between a scoping review and a systematic review in the introduction. The authors could explain why they chose a scoping review analysis in the discussion.*

Thank you for your suggestion. The sentence has been removed from the Introduction and a small paragraph has been added in the discussion section. *MATERIAL AND METHODS

Lines 170-172: During the data extraction it is very important to know the different quality of the studies evaluated in order to give more emphasis to the high quality studies as well as to show the current gaps in scientific literature.*

Even if it would be really interesting to analyse the quality of single studies, this was out of the scope of the present review. Unfortunately, the nature of the published papers did not allow us to perform a systematic review (for which a risk of bias assessment is a requirement) and/or to have a homogenous assessment of the quality of single studies.

*RESULTS

Lines 287-288: This result has not been sufficiently clarified, references 11 and 12 are quite different in their methodology and results, hence it should be analysed more in depth.*

Thank you for your suggestion. We modified the text focusing on the outcome that was of most interest for us, meaning the statistical difference among BIS values at clinically relevant DoA levels (identified in all the studies as MAC and MAC-multiples).

*Lines 288: The studies cited, references 11-12-14-16, are not so similar, therefore this statement appears to be incomplete. BIS was compared to different variables with different results and it should be explained accurately.*
Linked to the previous comment: we slightly modified the text to let the reader understand that we took in consideration only the outcome that was in common among studies and of interest for us (statistical difference among DoA levels, identified with MAC and MAC-multiples).

*DISCUSSION

Lines 364: Perhaps the results about using IOC and PSI indexes should be expressed in detail and discussed.*

Thank you for spotting it. A sentence has been added to provide further information.

*Lines 365-371: The BIS method is evaluated in 8 studies but the authors only discuss 5 out 8; they should also discuss the remaining three studies. Besides, the authors could give some possible reasons for the contrasting results when using the BIS method.*

Thank you for your suggestion. We agree with you; the other three studies have been included in the discussion. Possible reasons for the heterogenicity of the results and the unsuitability of BIS in pigs are discussed in the following lines.
